# Neurosecretory Protein GL Induces Fat Accumulation in Chicks

**DOI:** 10.3389/fendo.2019.00392

**Published:** 2019-06-19

**Authors:** Kenshiro Shikano, Eiko Iwakoshi-Ukena, Masaki Kato, Megumi Furumitsu, George E. Bentley, Lance J. Kriegsfeld, Kazuyoshi Ukena

**Affiliations:** ^1^Laboratory of Neuroendocrinology, Graduate School of Integrated Sciences for Life, Hiroshima University, Higashihiroshima, Japan; ^2^Department of Neurophysiology, Faculty of Medicine, Oita University, Yufu, Japan; ^3^Department of Integrative Biology, Helen Wills Neuroscience Institute, University of California, Berkeley, Berkeley, CA, United States; ^4^Department of Psychology, Helen Wills Neuroscience Institute, University of California, Berkeley, Berkeley, CA, United States

**Keywords:** neurosecretory protein, chicken, hypothalamus, fat accumulation, growth

## Abstract

We recently found a previously unidentified cDNA in chicken hypothalamus which encodes the precursor for neurosecretory protein GL (NPGL). A previous study showed that intracerebroventricular (i.c.v.) infusion of NPGL caused body mass gain in chicks. However, it was not clear which part(s) of the body gained mass. In the present study, we investigated which tissues increased in mass after chronic i.c.v. infusion of NPGL in chicks. We found that NPGL increased the masses of the liver, abdominal fat, and subcutaneous fat, while NPGL did not affect the masses of muscles, including pectoralis major, pectoralis minor, and biceps femoris. Oil Red O staining revealed that fat deposition had occurred in the liver. In addition, the size of the lipid droplets in the abdominal fat increased. Furthermore, we found an upregulation of lipogenesis and downregulation of lipolysis in the abdominal fat, but not in the liver. These results indicate that NPGL is involved in fat storage in chicks.

## Introduction

The processes of animal development and growth are regulated by several hormones, genetics, nutrition, and the environment (1). Among these factors, growth hormone (GH), produced by the pituitary gland, and insulin-like growth factor-1 (IGF-1), made by the liver, play important roles in the growth of peripheral tissues, including bone, liver, muscle, and adipose tissue (1, 2). In addition, increases in the masses of peripheral tissues are also influenced by energy states, such as starvation and satiation. For energy homeostasis, bioactive substances, including neuropeptide Y (NPY), glucocorticoids, and leptin from the hypothalamus, adrenal gland, and adipose tissue, can also influence body mass growth in mammals (3–5). These neuropeptides, or circulating steroids and peptide hormones are secreted in response to physiological conditions, and the signals are finally integrated in the hypothalamus to regulate feeding behavior. Owing to the complexity of feeding regulation and growth, the exact mechanism(s) regulating these physiological processes remains to be elucidated.

The efficiency of nutrient utilization and growth rate in domestic animals is very important for the farming/food industry. Particularly significant progress has been made over the last several decades in poultry production following genetic selection (6). Orexigenic and anorexigenic factors act differently in birds as compared to mammals. In mammals, NPY, agouti-related protein (AgRP), melanin-concentrating hormone (MCH), orexin, and ghrelin stimulate food intake (7, 8). In contrast to mammals, MCH and orexin do not affect food intake of chicks, and ghrelin inhibits feeding behavior (9). In addition to the differential effects in birds and mammals, it is likely that other undiscovered factors take part in the regulation of feeding and growth.

In a preceding study, we aimed to find unknown regulatory substance(s) in the chicken hypothalamus that affect neuronal control of feeding behavior and/or growth. Food intake, energy consumption, and increase in body mass are inextricably linked to animal growth. We recently deduced a cDNA from chicken hypothalamus that encodes the unidentified precursor to a small neurosecretory protein (10). The deduced pre-pro-protein contains a signal peptide sequence, a mature secretory protein sequence of 80 amino acid residues, a glycine amidation signal, and a cleavage signal consisting of dibasic amino acids (Lys-Arg) (10). The predicted C-terminal amino acid sequences were Gly-Leu-NH_2_, so we termed the protein neurosecretory protein GL (NPGL) (10). *In situ* hybridization revealed that the *NPGL* mRNA was expressed in the infundibular nucleus (IN) and the medial mammillary nucleus (MM) of the hypothalamus (10). The IN and MM in chicks are known to correspond to the mammalian arcuate nucleus (Arc) and the tuberomammillary nucleus (TMN), respectively; both nuclei are recognized to be involved in regulation of food intake in mammals. In addition, we found that the expression levels of *NPGL* mRNA were elevated during the post-hatch period (10). Furthermore, we reported that subcutaneous administration of NPGL for 4 days increased body mass gain independently of feeding behavior in chicks (10). Chronic intracerebroventricular (i.c.v.) infusion of NPGL for 2 weeks stimulated intake of food and water, with an associated rise in body mass (11). These data indicate that NPGL may influence growth processes of chicks. However, the specific body tissue(s) that are actually impacted by NPGL administration have not been elucidated. In the present study, we addressed this gap in our knowledge of NPGL action.

## Materials and Methods

### Animals

Male layer chicks (*Gallus domesticus*, 1 day old, *n* = 8 in each group) were obtained from a commercial hatchery (Nihon Layer, Gifu, Japan) and kept in a windowless room at 28°C on a light/dark cycle: 20 h light (4:00–24:00) and 4 h dark (0:00–4:00) according to our previous report (11). The chicks had access to food and water *ad libitum*. Chicks were raised in a group and then separated into individual cages from 4 days of age in order to measure individual food intake. The experimental protocols were in accordance with the Guide for the Care and Use of Laboratory Animals prepared by Hiroshima University (Higashi-Hiroshima, Japan).

### Production of Chicken NPGL

Chicken NPGL was synthesized with Fluorenylmethyloxycarbonyl (Fmoc) chemistry using a peptide synthesizer (Syro Wave; Biotage, Uppsala, Sweden) according to our previous method (12). The protein was cleaved from the resin with reagent K (trifluoloacetic acid: TFA 82.5%, phenol 5%, thioanisol 5%, H_2_O 5%, and 1,2-ethanedithiol 2.5%) for 3 h. The crude protein was purified by reverse-phase high-performance liquid chromatography (HPLC) using a C18 column (YMC-Pack ProC18, 10 × 150 mm; YMC, Kyoto, Japan) at a flow rate of 1.0 ml/min for 40 min with a linear gradient of 40–60% acetonitrile containing 0.1% TFA. The solvent was evaporated and lyophilized. The protein was dissolved in dimethyl sulfoxide (DMSO) and then diluted to a final concentration of 0.5 mM glutathione disulfide, 5 mM glutathione, 50% acetonitrile, 1 mM EDTA, 10% DMSO, 0.4 M Tris-HCl (pH 8.5). The solvent was rotated for 2 days at room temperature to allow for an intramolecular disulfide bond to form, purified by HPLC and then lyophilized. The purity of the protein was >95%. Lyophilized NPGL was weighed using an analytical and precision balance (AP125WD; Shimadzu, Kyoto, Japan).

### i.c.v. Infusion of NPGL for 2 Weeks

NPGL was dissolved in absolute propylene glycol and adjusted to 30% propylene glycol as a vehicle solution. Eight day old chicks were i.c.v. infused with 0 (vehicle) or 15 nmol/day NPGL via an infusion cannula (model 328OP; Plastics One, Roanoke, VA) and an Alzet mini-osmotic pump (model 2002, delivery rate 0.5 μl/h; DURECT Corporation, Cupertino, CA). The dose was determined on the basis of previous studies (11, 13). The infusion cannula tip was implanted into the lateral ventricle: 2.0 mm rostral to lambda, 1.0 mm lateral to midline, and 5.5 mm ventral to the skull surface. Osmotic mini-pumps containing vehicle or NPGL were implanted subcutaneously in the neck according to our previous method (11).

Body mass and food intake were measured daily (between 9:00–10:00) throughout the experiment. After 2 weeks of i.c.v. infusion of NPGL, chicks were killed by decapitation and masses of liver, abdominal fat, subcutaneous fat, pectoralis major muscle, pectoralis minor muscle, and biceps femoris muscle were measured.

### Histological Analysis

For Oil Red O staining to detect fat accumulation, the livers from 5 animals of each group were fixed in 4% paraformaldehyde and sliced into 10 μm sections. These were air-dried, rinsed with 60% isopropanol, stained with Oil Red O solution for 15 min at 37°C, and rinsed with 60% isopropanol. The nucleus was counterstained with hematoxylin for 5 min, and the sections were washed in tap water. The slides were mounted in aqueous mounting medium for microscopic examination.

For hematoxylin and eosin staining, fixed abdominal fat was embedded in paraffin and cut into 7 μm sections with a microtome. The sections were then delipidated with acetone. The nucleus and cytoplasm were stained with hematoxylin and eosin (5 min for each stain), and the sections were washed in tap water. After serial dehydration with alcohol and clearing with xylene, the sections were mounted on slides for microscopic examination using an Eclipse E600 conventional microscope (Nikon, Tokyo, Japan).

### Real-Time RT-PCR

The procedures were carried out in a similar manner to our previous report (11). At the end of the NPGL infusions, chicks were killed by decapitation between 13:00–15:00. The liver and abdominal fat were dissected out and snap-frozen in liquid nitrogen. RNA was extracted using TRIzol reagent for liver (Life Technologies, Carlsbad, CA) or QIAzol lysis reagent for abdominal fat (QIAGEN, Venlo, Netherlands) following the manufacturer's instructions. First-strand cDNA was synthesized from total RNA (1 μg) using a ReverTra Ace kit (TOYOBO, Osaka, Japan).

PCR amplifications were conducted using THUNDERBIRD SYBR qPCR Mix (TOYOBO) and the following procedure: 95°C for 20 s, followed by 40 cycles at 95°C for 3 s, and at 60°C for 30 s using a real-time thermal cycler (CFX Connect; BioRad, Hercules, CA). Amplifications of lipogenic and lipolytic enzymes and related factors were performed with the primer sets listed in Table 1. We chose acetyl-CoA carboxylase (ACC), fatty acid synthase (FAS), stearoyl-CoA desaturase 1 (SCD1), malic enzyme (ME) as lipogenic enzymes, peroxisome proliferator-activated receptor γ (PPARγ) as cell differentiation marker, fatty acid transporter 1 (FATP1) as fatty acid uptake, and PPARα, carnitine palmitoyltransferase 1a (CPT1a), lipoprotein lipase (LPL), adipose triglyceride lipase (ATGL), and comparative gene identification-58 (CGI-58) as lipolytic enzymes and related factors.

**Table 1:** Sequences of oligonucleotide primers for real-time PCR.

**Gene**	**Forward primer**	**Reverse primer**	**Accession no**.
*ACC*	AATGGCAGCTTTGGAGGTGT	TCTGTTTGGGTGGGAGGTG	NM_205505.1
*FAS*	CCAACGATTACCCGTCTCAA	CAGGCTCTGTATGCTGTCCAA	NM_205155.2
*SCD1*	AGTGGTGTTGCTGTGCTTCA	CTAAGGTGTAGCGCAGGATG	NM_204890.1
*ME*	AGTGCCTACCTGTGATGTTG	GGCTTGACCTCTGATTCTCT	NM_204303.1
*PPARγ*	TCAAGCATTTCTTCACCACACT	ATTGCACTTTGGCAATCCTGG	NM_001001460.1
*FATP1*	TACAATGTGCTCCAGAAGGG	GTCTGGTTGAGGATGTGACTC	NM_001039602.2
*PPARα*	TGCTGTGGAGATCGTCCTGGT	AGAGGAAGATATCGTCAGGATGG	NM_001001464.1
*CPT1a*	TGATCTGAAGAAGAACCCTGAGAT	TCCAAAGCGATGAGAATCCG	NM_001012898.1
*LPL*	CAGTGCAACTTCAACCATACCA	AACCAGCCAGTCCACAACAA	NM_205282.1
*ATGL*	CACTGCCATGATGGTCCCCTA	CCACAAGGAGATGCTGAAGAA	NM_001113291.1
*CGI-58*	ACCGTGGTTTATGGAGCACG	GAAACAGTGTGCAAACAGAGCC	NM_001278145.1
*ACTB*	CCAGAGTCCATCACAATACC	AGCCAACAGAGAGAAGATGA	NM_205518.1

The relative quantification for each expression was determined by the 2^−ΔΔCt^ method (14) using β-actin (ACTB) as the internal control.

### Statistical Analysis

Data were analyzed with Student's *t*-test for tissue mass and mRNA expression or one-way repeated measures analysis of variance (ANOVA) followed by Bonferroni's test for body mass gain and cumulative food intake. The significance level was set at *P* < 0.05. All results are expressed as the mean ± SEM.

## Results

### Effect of i.c.v. Infusion of NPGL on Body Mass Gain, Food Intake, and Body Composition

**Figure 1:**
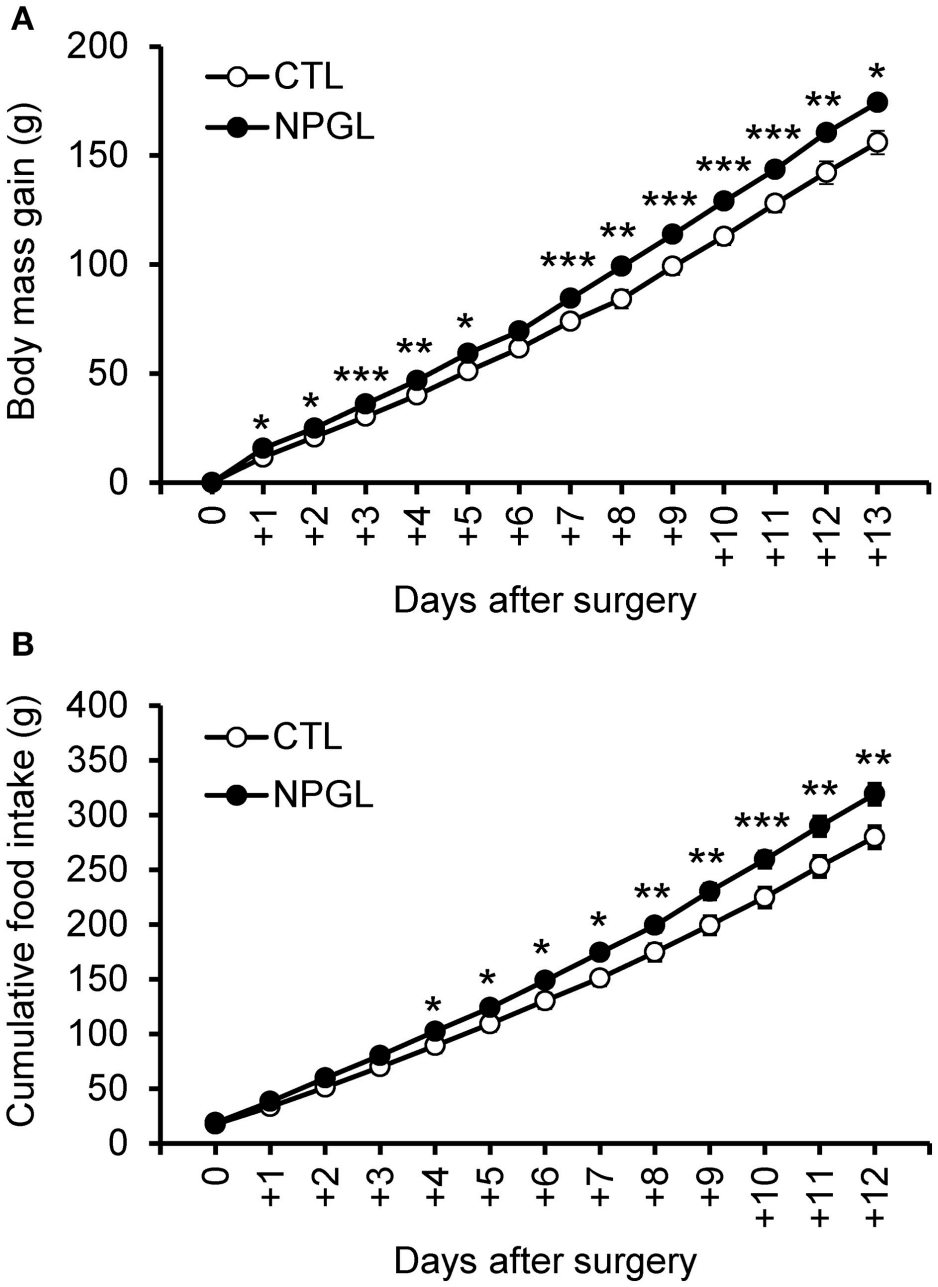
Effect of chronic i.c.v. infusion of NPGL on body mass gain and food intake. The results were obtained by the infusion of the vehicle (control; CTL) and NPGL. The change in the body mass gain after surgery **(A)**. The cumulative food intake **(B)**. Data are expressed as the mean ± SEM (*n* = 8). Data were analyzed with a one-way repeated measures analysis of variance (ANOVA), followed by Bonferroni's test. An asterisk indicates a statistically significant difference (^*^P < 0.05, ^**^P < 0.01, ^***^P < 0.001).

**Figure 2:**
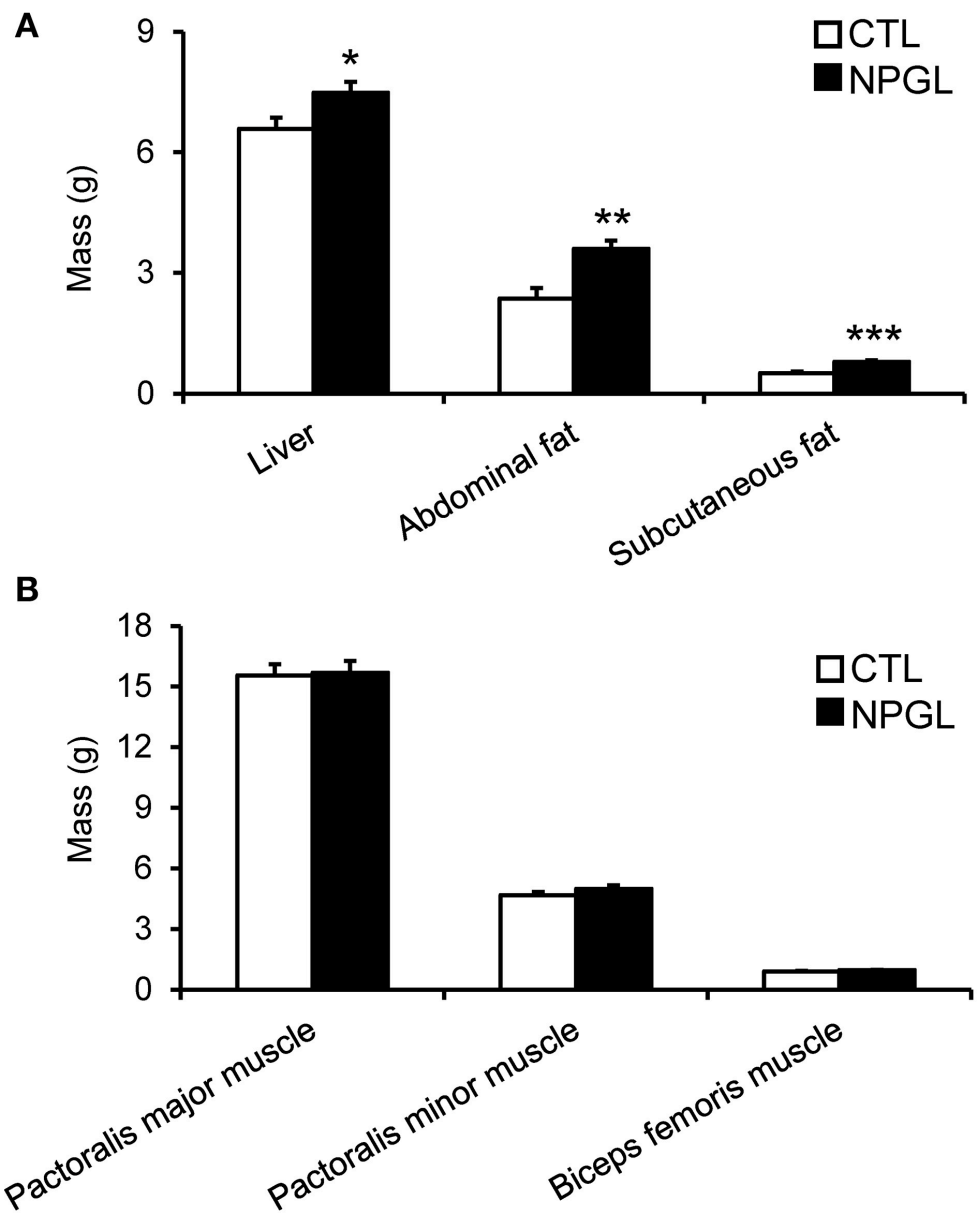
Effect of chronic i.c.v. infusion of NPGL on body composition. The results were obtained by the infusion of the vehicle (CTL) and NPGL for 2 weeks. The masses of the liver, abdominal fat, and subcutaneous fat **(A)**, and pectoralis major muscle, pectoralis minor muscle, and biceps femoris muscle **(B)** were measured at the end of experiment. Data are expressed as the mean ± SEM (*n* = 8). Data were analyzed by Student's *t*-test. Asterisks indicate statistically significant differences (^*^P < 0.05, ^**^P < 0.01, ^***^P < 0.001).

NPGL infusion significantly promoted body mass gain (Figure 1A), and NPGL also increased cumulative food intake (Figure 1B). Furthermore, NPGL increased the mass of the liver, abdominal fat, and subcutaneous fat (Figure 2A), while no change was observed in the muscle mass of the pectoralis major muscle, pectoralis minor muscle, and biceps femoris muscle (Figure 2B).

### Effect of i.c.v. Infusion of NPGL on Lipid Deposition in the Liver and Abdominal Fat

**Figure 3:**
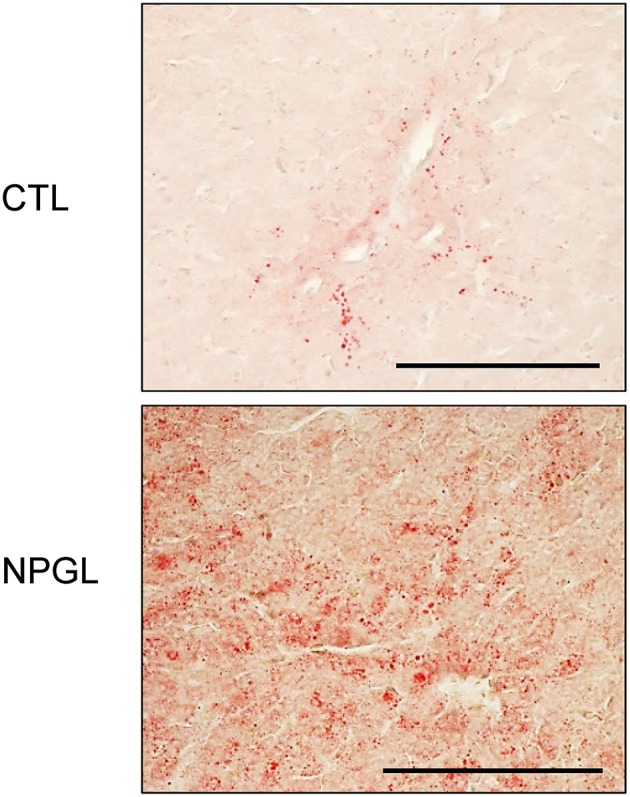
Effect of chronic i.c.v. infusion of NPGL on lipid deposition in the liver. The results were obtained 2 weeks after infusion of the vehicle (CTL) and NPGL. Representative photomicrographs of the sections stained by Oil Red O staining (scale bar = 100 μm).

**Figure 4:**
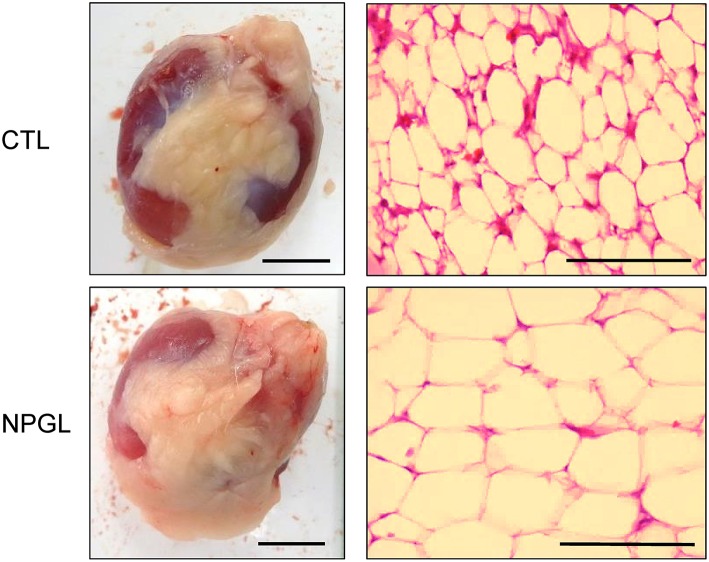
Effect of chronic i.c.v. infusion of NPGL on the lipid droplets in the abdominal fat. The results were obtained 2 weeks after infusion of the vehicle (CTL) and NPGL. Exterior photographs of the abdominal fat around the gizzard (left panel, scale bar = 1 cm), and representative photographs of the sections stained by hematoxylin and eosin staining (right panel, scale bar = 100 μm).

Lipid droplets were observed in the liver after chronic i.c.v. infusion of NPGL, increasing its mass (Figure 3). Moreover, abdominal fat accumulated around the gizzard in the NPGL-infused chicks (Figure 4, left in lower panel). The lipid droplets in the abdominal fat of the chicks were substantially larger after chronic NPGL infusion than those in the controls (Figure 4, right in lower panel).

### Effect of i.c.v. Infusion of NPGL on the mRNA Expression of Lipogenic and Lipolytic Factors in the Liver and Abdominal Fat

**Figure 5:**
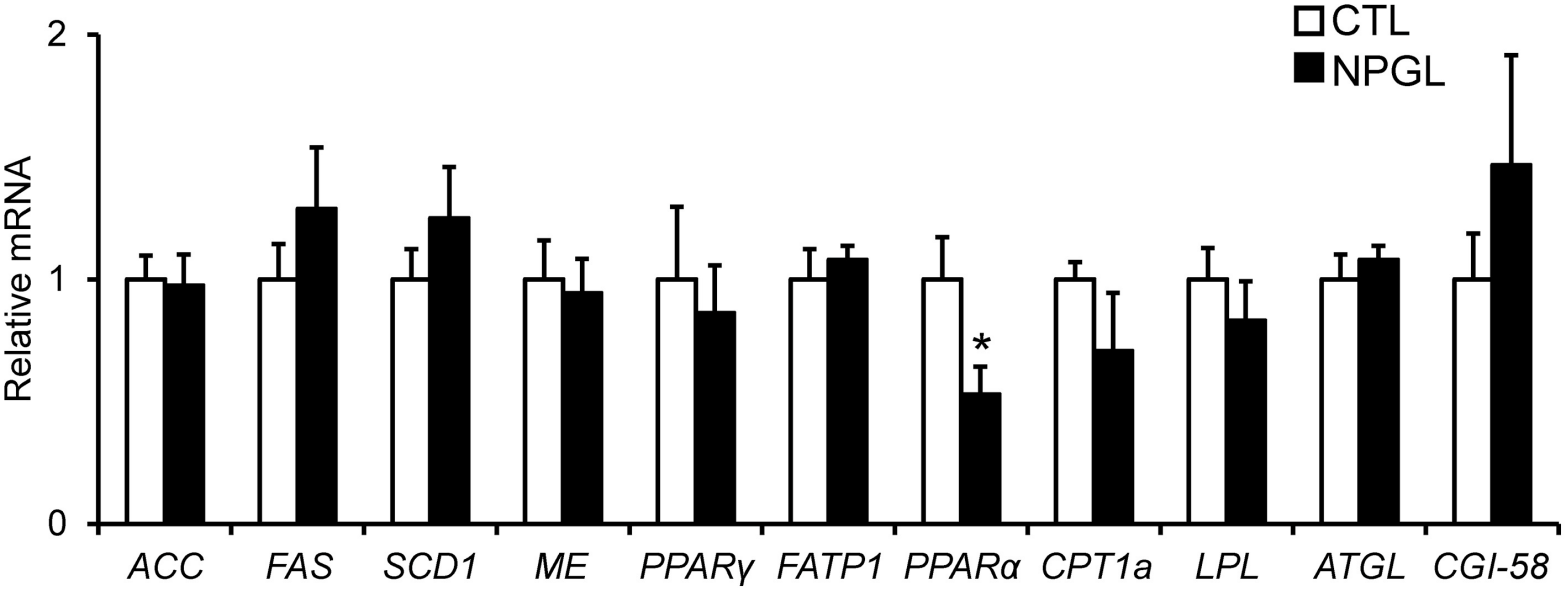
Effect of chronic i.c.v. infusion of NPGL on the mRNA expression of lipogenic and lipolytic factors [acetyl-CoA carboxylase (*ACC*), fatty acid synthase (*FAS*), stearoyl-CoA desaturase 1 (*SCD1*), malic enzyme (*ME*), peroxisome proliferator-activated receptor γ (*PPAR*γ), fatty acid transporter 1 (*FATP1*), peroxisome proliferator-activated receptor α (*PPAR*α), carnitine palmitoyltransferase 1a (*CPT1a*), lipoprotein lipase (*LPL*), adipose triglyceride lipase (*ATGL*), and comparative gene identification-58 (*CGI-58*)] in the liver. The results were obtained 2 weeks after infusion of the vehicle (CTL) and NPGL. Data are expressed as the mean ± SEM (*n* = 8). Data were analyzed by Student's *t*-test. An asterisk indicates a statistically significant difference (^*^P < 0.05).

**Figure 6:**
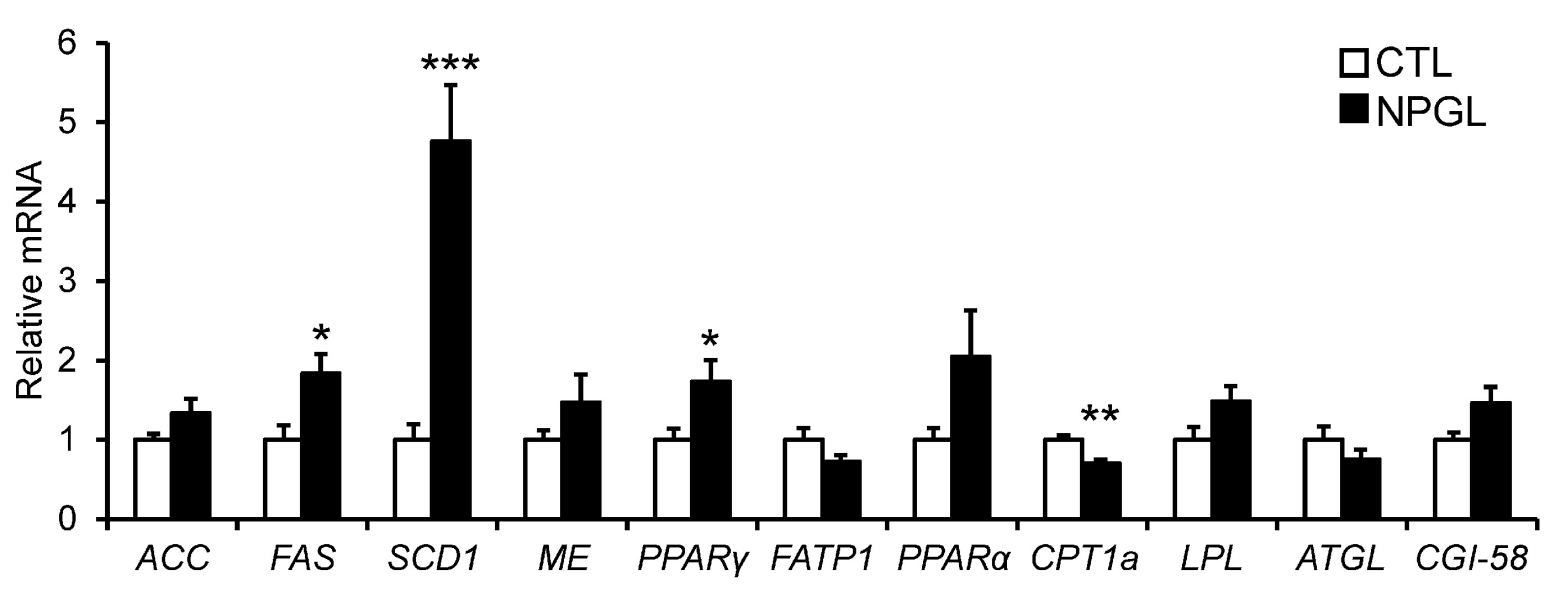
Effect of chronic i.c.v. infusion of NPGL on the mRNA expression of lipogenic and lipolytic factors [acetyl-CoA carboxylase (*ACC*), fatty acid synthase (*FAS*), stearoyl-CoA desaturase 1 (*SCD1*), malic enzyme (*ME*), peroxisome proliferator-activated receptor γ (*PPAR*γ), fatty acid transporter 1 (*FATP1*), peroxisome proliferator-activated receptor α (*PPAR*α), carnitine palmitoyltransferase 1a (*CPT1a*), lipoprotein lipase (*LPL*), adipose triglyceride lipase (*ATGL*), and comparative gene identification-58 (*CGI-58*)] in abdominal fat. The results were obtained 2 weeks after infusion of the vehicle (CTL) and NPGL. Data are expressed as the mean ± SEM (*n* = 8). Data were analyzed by Student's *t*-test. Asterisks indicate statistically significant differences (^*^P < 0.05, ^**^P < 0.01, ^***^P < 0.001).

To investigate the gene expression of lipogenic and lipolytic factors in the liver and abdominal fat, we analyzed the mRNA levels of *ACC, FAS, SCD1, ME, PPAR*γ, *FATP1, PPAR*α, *CPT1a, LPL, ATGL*, and *CGI-58* after chronic i.c.v. infusion of NPGL. NPGL decreased the mRNA expression of *PPAR*α in the liver (Figure 5) and increased the expression of *FAS, SCD1*, and *PPAR*γ in the abdominal fat; whereas, NPGL decreased the *CPT1a* mRNA (Figure 6).

## Discussion

The precursor gene for NPGL was identified in the chicken hypothalamus through a cDNA subtractive screen designed to identify novel neuronal substance(s) in the avian brain (10). After sequencing 596 clones from hypothalamic cDNA, we found an unidentified cDNA that encodes an unknown protein. The deduced precursor protein consisted of 182 amino acid residues, containing a putative secretory protein of 80 amino acid residues, and which had Gly-Leu-NH_2_ at its C-terminus. Therefore, the protein was termed neurosecretory protein GL (NPGL) (10). As NPGL contains two Cys residues, the presence of an intramolecular disulfide bond bridge is likely (10). Subcutaneous administration of NPGL can cause an increase in body mass of chicks without affecting feeding behavior (10). However, the exact body components that are affected by NPGL had not been studied. The aim of this study was determination of the body component(s) that increased after NPGL infusion. We measured the masses of various tissues, including the liver, fat, and muscles after 2 weeks of i.c.v. NPGL infusion. The results showed that NPGL increased the mass of the liver, abdominal fat, and subcutaneous fat (Figure 2A), while no change was observed in the muscle masses of pectoralis major, pectoralis minor, and biceps femoris (Figure 2B). As we speculated that NPGL stimulates fat accumulation in the liver and adipose tissue, this phenomenon was confirmed using a morphological analysis. The results revealed lipid deposition in the liver and the enlargement of lipid droplets in the abdominal fat (Figures 3, 4).

In the experiments to survey the gene expression related to lipogenic and lipolytic factors, we found a decrease in the *PPAR*α mRNA in the liver, increases in *FAS, SCD1*, and *PPAR*γ, and a reduction of *CPT1a* in the abdominal fat. It is known that target genes of PPARα participate in fatty acid β-oxidation in the liver (15, 16). FAS and SCD1 are lipogenic enzymes, and CPT1a is lipolytic enzyme (17). It has been reported that PPARγ plays important roles in the process of adipogenic differentiation (18). Taken together with these findings, the present study suggests that NPGL inhibits fatty acid oxidation in the liver and induces *de novo* lipogenesis in adipose tissue. It has been demonstrated previously in birds that *de novo* fatty acid synthesis takes place predominantly in the liver (19). The present data indicate the novel possibility of *de novo* lipogenesis in the adipose tissue of birds.

In mammals, *de novo* lipogenesis is the process of transforming non-lipid precursors into fatty acids and triglycerides for energy storage (20). Although *de novo* lipogenesis in adipose tissue is important for the maintenance of metabolic homeostasis, *de novo* lipogenesis in non-adipose tissues, such as the liver and muscle, results in ectopic lipid deposition, lipotoxicity, and metabolic stress disorder (21, 22). Lipogenesis and lipolysis in adipose tissue are controlled by the hypothalamus via the sympathetic nervous system (23, 24). Therefore, it is likely that NPGL takes part in the sympathetic control of fat accumulation in chicks, although there may be other pathways that mediate its actions. Future studies are necessary to classify the target regions and neuronal networks regarding the NPGL neurons to elucidate the exact mechanism of *de novo* lipogenesis in birds.

The present study also showed that i.c.v. infusion of NPGL stimulated food intake. As mentioned above, subcutaneous infusion of NPGL did not affect the feeding behavior of chicks (10). The results from the present study suggest that NPGL may act on the brain to stimulate feeding behavior, although we have found that NPGL did not change the mRNA expression of various hypothalamic ingestion-related neuropeptides, i.e., NPY, AgRP, proopiomelanocortin (POMC; precursor of α-melanocyte-stimulating hormone), glucagon-like peptide (GLP-1), and cholecystokinin (CCK) (11). Future studies are needed to elucidate the target neurons of NPGL that alter feeding behavior.

After the identification of the NPGL precursor gene in the chicken hypothalamus, we searched for related genes in the genome database (TBLASTN) using the amino acid sequence of the NPGL precursor. We found an orthologous gene in humans and rats (10). In addition, the NPGL precursor gene is conserved in mouse, turtle, frog, and fish (25). In fact, we cloned the cDNA encoding NPGL in the mouse and rat hypothalamus and found that the mature NPGL amino acid sequence was 85% similar between chicken and rodent (26, 27). In mice, we demonstrated that acute i.c.v. injection of NPGL increased food intake from 2 to 10 h after administration (26). Although we also performed acute injection of NPGL in chicks in our preliminary experiments, we did not observe a significant effect on feeding behavior. The data suggest that the mechanisms of action of NPGL on food intake differ between chicks and mice. The present data show that chronic infusion of NPGL stimulated feeding behavior as mentioned above. We need to elucidate the reasons for differential effects of acute and chronic administration of NPGL on food intake in future studies.

Recently, we also investigated the biological actions of NPGL in rats by overexpressing the NPGL precursor gene in the hypothalamus and chronic i.c.v. infusion of NPGL peptide, similar to the present study. The results showed that NPGL induced a significant rise in the mass of adipose tissue and the magnitude of adipocytes in rats (27). Next, we investigated the mRNA expression of lipogenic and lipolytic enzymes in the adipose tissue and liver of rats and found that the mRNA expression levels of the lipogenic enzymes in the adipose tissue significantly increased after *NPGL* overexpression and i.c.v. infusion of NPGL, but no differences were detected in the liver (27). Thus, NPGL-induced *de novo* lipogenesis does not occur in the liver, but is restricted to adipose tissue. To our knowledge, the previous study in rats was the first report of an endogenous neuronal substance that can regulate peripheral *de novo* lipogenesis in animals (27). In the present study, we also found that peripheral *de novo* lipogenesis was induced by chronic i.c.v. infusion of NPGL in chicks. These results suggest that NPGL-induced *de novo* lipogenesis in adipose tissue is a conserved property in birds and rodents.

In conclusion, a chronic i.c.v. administration of NPGL stimulated food intake, increased the masses of the liver and adipose tissue, and finally, caused an increase in body mass gain in developing chicks. Thus, NPGL is a positive regulator of feeding and growth post-hatch. This is the first report describing the upregulation of *de novo* lipogenesis from chronic i.c.v. infusion of NPGL in birds. The cognate receptor for NPGL has yet to be characterized in any animal, but this will be necessary in order to elucidate the precise roles of NPGL action in the brain. Regulation of feeding behavior and fat storage are vital for survival and for the transition into specific life-history stages, such as pregnancy, puberty, aging, migration, and hibernation. Future comparative analyses using other animal models will help elucidate the unity and diversity of the physiological functions of NPGL.

## Ethics Statement

The experimental protocols were in accordance with the Guide for the Care and Use of Laboratory Animals prepared by Hiroshima University (Higashi-Hiroshima, Japan).

## Author Contributions

KU conceived and designed the experiments. All authors performed the experiments and analyzed the data. KU, KS, EI-U, and GB wrote the paper.

### Conflict of Interest Statement

The authors declare that the research was conducted in the absence of any commercial or financial relationships that could be construed as a potential conflict of interest.
